# Comparing radiation exposure during percutaneous vertebroplasty using one- vs. two-fluoroscopic technique

**DOI:** 10.1186/1471-2474-14-38

**Published:** 2013-01-22

**Authors:** Yen-Yao Li, Tsung-Jen Huang, Chin-Chang Cheng, Meng-Huang Wu, Ching-Yu Lee

**Affiliations:** 1 Department of Orthopedic Surgery, Chang Gung Memorial Hospital, Chiayi, No.6, W. Sec., Chia-pu Rd, Putz City, Chiayi County, 613, Taiwan; 2 College of Medicine, Chang Gung University, Taoyuan, Taiwan

**Keywords:** Vertebral compression fracture, Osteoporosis, Vertebroplasty, Radiation dose

## Abstract

**Background:**

Percutaneous vertebroplasty (PV) requires relatively lengthy fluoroscopic guidance, which might lead to substantial radiation exposure to patients or operators. The two-fluoroscopic technique (two-plane radiographs obtained using two fluoroscopes) during PV can provide simultaneous two-planar projections with reducing operative time. However, the two-fluoroscopic technique may expose the operator or patient to increased radiation dose. The aim of this study was to quantify the amount of radiation exposure to the patient or operator that occurs during PV using one- vs. two-fluoroscopic technique.

**Methods:**

Two radiation dosimeters were placed on the right flank of each patient and on the upper sternum of each operator during 26 single-level PV procedures by one senior surgeon. The use of two-fluoroscopic technique (13 patients) and one-fluoroscopic technique (13 patients) were allocated in a consecutive and alternative manner. The operative time and mean radiation dose to each patient and operator were monitored and compared between groups.

**Results:**

Mean radiation dose to the patient was 1.97 ± 1.20 mSv (95% CI, 0.71 to 3.23) for the one-fluoroscopic technique group vs. 0.95 ± 0.34 mSv (95% CI, 0.85 to 1.23) for the two-fluoroscopic technique group (*P* =0.031). Mean radiation dose to the operator was 0.27 ± 0.12 mSv (95% CI, 0.17–0.56) for the one-fluoroscopic technique group vs. 0.25 ± 0.14 mSv (95% CI, 0.06–0.44) for the two-fluoroscopic technique group (*P* = 0.653). The operative time was significantly different between groups: 47.15 ± 13.48 min (range, 20–75) for the one-fluoroscopic technique group vs. 36.62 ± 8.42 min (range, 21–50) for the two-fluoroscopic technique group (*P* =0.019).

**Conclusion:**

Compared to the one-fluoroscopic technique, the two-fluoroscopic technique used during PV provides not only shorter operative times but also reduces the radiation exposure to the patient. There was no significant difference between the two techniques with regards to radiation exposure to the operator.

## Background

Minimally invasive procedures in spinal surgery have become increasingly popular during the past decade. While the reduction in soft tissue trauma and more rapid recovery of the patient are unquestionable assets attributable to these techniques, the relative risk to the patient and the operator from the considerable radiation exposure during percutaneous vertebroplasty (PV) requires further evaluation.

PV is a minimally invasive spinal procedure that has been safely performed using various imaging modalities including C-arm fluoroscopy, biplane fluoroscopy, and computed tomography [1-3]. Our earlier reports detailed the use of two sets of C-arm fluoroscopes during PV which provided two-plane radiograms in the anteroposterior (AP) and lateral views [4]. During the two-fluoroscopic technique, however, the operator or patient may undergo considerable radiation exposure.

The aim of this study was to quantify the amount of radiation exposure to the patient or operator that occurs during PV. We conducted a prospective trial in which we measured the radiation dose to the patient and operator during PV using two techniques, the two C-arm fluoroscopic technique and the one C-arm fluoroscopic technique.

## Methods

### Subjects

The study was approved by the Ethics Committee and the Institutional Review Board of the authors’ institution (IRB reference number: 97-1931A3). Twenty-six consecutive patients who underwent a single-level PV by a single senior surgeon were enrolled in the study. Informed consent was obtained from all patients. The patients were assigned alternately to undergo PV using either the two C-arm fluoroscopic technique (13 patients) or the one C-arm fluoroscopic technique (13 patients).

### Dosimetry

**Figure 1:**
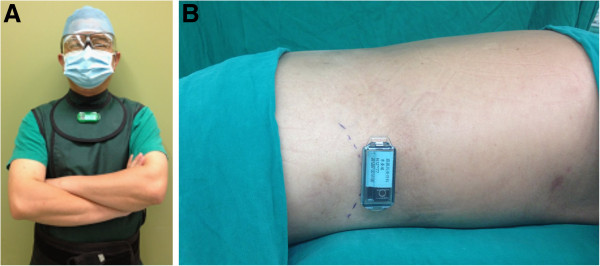
**Thermoluminescent dosimeter.** (**A**) Operator’s dosimeter placement at the upper sternum (juxta-thyroid). (**B**) Patient’s dosimeter placement at right flank proximal to iliac crest (the dotted line).

Thermoluminescent dosimeters (Becquerel & Sievert, Taipei, Taiwan) were placed on the right flank of each patient (proximal to the iliac crest) and the upper sternum (juxta-thyroid) of the operator during the PV procedures (Figure 1). Use of a thermoluminescent dosimeter (TLD) is potentially the most accurate way of measuring actual skin dose to the patient, however, it is often impossible to know exactly where on the skin the peak dose will be exposed during vertebroplasty. Ideally, using two TLDs in either plane we can measure patient exposure in both planes. However, it was impossible in the current study since the TLD is not sterile, which can not be placed in the surgical field. Generally, lateral radiographs should be taken more times than anteroposterior view to check cement leakage into spinal canal. In the study, so that, we use the TLD placed on the right flank of each patient to be close to the source of lateral fluoroscope.

The TLDs remained in place during the entire procedure including the first projection (which checked the index level for PV) and the last projection (following removal of the injection needle) and both dosimeters were subsequently removed for dose measurements.

### PV with two-fluoroscopic technique

**Figure 2:**
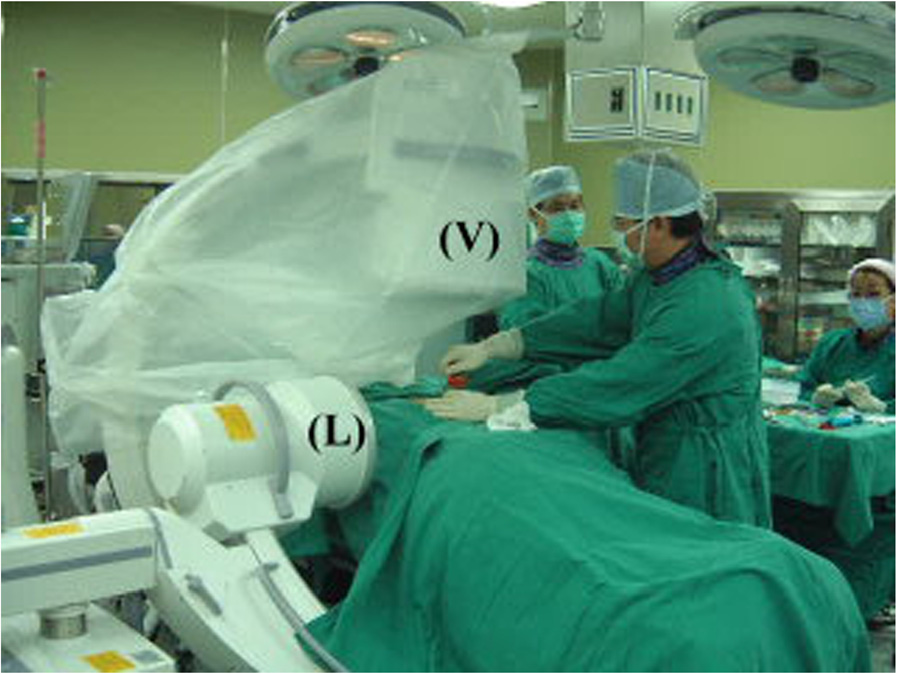
**Placement of two fluoroscopes.** The AP fluoroscope (V) is positioned vertically with the C-arm’s pivot maintained at 45° or less to the long axis of the operating table. The C-arm of the lateral fluoroscope (L) is placed under the operating table horizontally while it is tilted away from the C-arm of the AP fluoroscope to prevent collision.

This technique (Figure 2) is unique with regards to the positioning of the two fluoroscopes for the PV procedure. The two fluoroscopes (GE/OEC 9800, Salt Lake City, UT, USA) were placed on the same side, allowing the operator to perform the operation on the contralateral side. The pulsed fluoroscopy mode was adopted with 15 pulse per second. For all the procedures, the range of the source voltage was 40–110 kV and the current was 0.2-8 mA. Depending on the patient's position, the fluoroscope used for taking AP radiographs (AP-fluoroscope) was placed cephalad to the fluoroscope used for taking lateral radiographs (lateral-fluoroscope). The AP fluoroscope was positioned with the C-arm's pivot maintained at 45° or less to the long axis of the operating table. The C-arm of the lateral fluoroscope was placed under the operating table with the pivot vertical to the long axis of the operating table while the C-arm’s arch was tilted away from the source of the AP fluoroscope to prevent a collision. The AP and lateral projections were controlled by the operator via foot pedals without the need to turn the C-arm. The entry point of the spinal needle on the index pedicle was easily identified under the guidance of the two fluoroscopes.

### Data collection

The body mass index (BMI) of each patient and the vertebral level used for PV were recorded. The operative time was measured from the start of the local anesthesia to removal of the spinal needle after solidification of the delivered bone cement. The radiation dose (in mSv) to the patient and operator were recorded during the operation (Table 1).

**Table 1:** Comparison of radiation dose, operative time and body mass index between one- vs. two-fluoroscopic groups during percutaneous vertebroplasty

	**One-fluoroscopic technique**	**Two-fluoroscopic technique**	***P*-value**
Operative time (min)	47.15 ± 13.48	36.62 ± 8.42	0.019
T1 (*m*Sv)	0.27 ± 0.12	0.25 ± 0.14	0.653
T2 (*m*Sv)	1.97 ± 1.20	0.95 ± 0.34	0.031
BMI	24.71 ± 2.78	23.42 ± 2.88	0.204

*T1* Mean radiation dose to the operator, *T2* Mean radiation dose to the patient, *BMI* Body Mass Index.

### Statistical analysis

The power analysis carried out with the computer program GPower 3.1 software [5]. The power of each Wilcoxon-Mann–Whitney (two groups) unpaired test was determined by use of power analysis. A post hoc power analysis was performed with a sample size of n = 13 per group to examine the potential for type II errors in the data analysis. Assuming an alpha level of 0.05 with a two-sided alternative hypothesis, both the comparisons on radiation dose and operative time between using the two-fluoroscopic technique vs. using the one-fluoroscopic technique had at least 80% power to detect the minimum clinically important differences. Independent variables were compared between groups using a *Wilcoxon* test for two independent sample means. A p-value <0.05 was considered significant. We compared the mean radiation dose to the patient and the operator while using the two-fluoroscopic technique vs. using the one-fluoroscopic technique.

## Results

Twenty-six patients underwent PV, with 13 patients undergoing PV using the two-fluoroscopic technique and 13 using the one-fluoroscopic technique. Most of vertebral fractures were located at the thoracolumbar junction. There was no significant difference regarding BMI between groups (Table 1). The operative time was significantly different between groups with a shorter operative time observed using the two-fluoroscopic technique (36.62 ± 8.42 min) compared to the one-fluoroscopic technique (47.15 ± 13.48 min), *P =* 0.019 (Table 1).

The mean radiation dose to each patient using the two-fluoroscopic technique was 0.95 ± 0.34 mSv (95% CI, 0.85 to 1.23), compared with 1.97 ± 1.20 mSv using the one-fluoroscopic technique (95% CI, 0.71 to 3.23). The difference between groups was significant (*P* = 0.031) (Table 1). The mean radiation dose to the operator was not significantly different between two groups, although a less dose in two-fluoroscopic technique (95% CI, 0.25 ± 0.14 mSv) than the one- fluoroscopic technique (95% CI, 0.27 ± 0.12 mSv) was noted (Table 1).

## Discussion

**T**he PV procedure requires radiographic navigation in two planes (anteroposterior and lateral views) to identify the entry point for insertion of the spinal needle on the index vertebral pedicle(s). Furthermore, real-time fluoroscopic monitoring is usually recommended during cement injection during PV. With the popularity of PV, this technique draws concern regarding radiation exposure to the patient and operator. Several studies have investigated the radiation exposure during PV and found that the radiation-related risk may be considerable [6-9]. The National council in Radiation Protection and Measurements in 1993 suggested that the occupational exposure limits for extremity (eg. Hands) is 500 mSv/y, for the eye is 150 mSv/y, and for the total dose (whole body) is 50 mSv/y [10]. In our study, the whole body dose without protection is 0.27 and 0.25 mSv for the one-fluoroscopic technique and two-fluoroscopic technique, respectively. Therefore, if an operator did not wear the lead apron for shielding, the total exposure dose (whole body) will exceed the annual limit after 200 single-level vertebroplasty procedures annually regardless of one or two-fluoroscopic techniques. According to Mroz's study [11], the whole body dose (0.248 mSv/vertebra) to the operator during kyphoplasty is similar to that of our study. They also measured the exposure doses to the hand and concluded that the exposure dose to the hand would exceed the annual limit after 300 levels of kyphoplasty. Therefore they recommend the operator should always consider the appropriate protection (eg. lead gloves) against the radiation exposure to the hands. However, Wagner [12] et al. evaluated 4 different type protective gloves and reported a large variation in radiation attenuation from exposure reduction of 7% to almost 50%. So that, even though wearing protective gloves, the operator should place his or her hand as away from the path of the radiation beam as possible.

**T**he PV technique has rapidly evolved since Galibert performed the first PV in France in 1984 [13]. With respect to radiographic guidance, one-fluoroscopic technique or combined CT and fluoroscopic guidance were usually used to monitor PV [1]. We reported that the two-fluoroscopic technique [14] provides concurrent, real-time AP and lateral radiographic views and can reduce the operative time for PV. The technique is also more convenient since it allows the operator to take both AP and lateral projections without turning the C-arm. Mehdizade [15] et al. used biplane fluoroscopy unit to monitor vertebroplasty, which was similar to two-fluoroscopic technique in our study. They reported that the exposure doses to the operator were 0.022-3.256 mGy with the TLD outside and 0.01-0.47 mGy inside the lead apron. Those data seems to be comparable to our measurements as 0.11-0.39 mSv (0.25 ± 0.14 mSv) with two-fluoroscopic technique. However, we did not know the average dose in their study. Fitousi [16] et al. reported that the occupational exposure to the operator was 0.011 mGy in effective dose, 0.328 mGy in eye dose and 1.861 mGy in hand dose, during vertebroplasty with continuous fluoroscopy (Philips DVIS 3000). By using mobile shielding devices (eg. lead sheet), they found that the effective dose to the operator can be reduced by more than 75%.

**B**oszczyk [17] et al. evaluated the radiation exposure time (ET) of kyphoplasty using the two-fluoroscopic technique and reported that the ET was shorter compared to other studies using the one fluoroscopic technique [18]. Instead of ET, we evaluated radiation dose to the patient and found that the average dose (1.97 mSv) with the one-fluoroscopic technique was significantly higher compared to the dose measured during the two-fluoroscopic technique (0.95 mSv), *P* = 0.031. We agree with Boszczyk’s comment that once an optimal setting for the two-fluoroscopic technique has been found, it is maintained throughout the procedure and no radiation is “wasted” during readjustment for the other plane film. Furthermore, in the current study, the mean operative time using the two-fluoroscopic technique was shorter than that found using the one-fluoroscopic technique. The reduced operative time with the two-fluoroscopic technique may have resulted from the time saved by not having to readjust the C-arm to the other plane compared to the time used during the one-fluoroscopic technique in readjusting the X-ray generator to the appropriate position.

**I**n our study, the exposure doses to the patient are significantly different (*P* = 0.031) between using one-fluoroscopic and two-fluoroscopic technique. However, the doses to the operator are not different statistically. The dosimeter on the operator is mainly exposed to the scatter radiation, while that on the patient is partly exposed to primary radiation. Therefore, the dose amount on the operator is less than that on the patient (1.97, 0.95 mSv to patient Vs.0.27, 0.25 mSv to operator). Probably, for the small dose amount to the operator, we need large sample size to prove the doses to the operator different between the two techniques, if any.

**T**he mean radiation dose to the operator per PV level was lower compared with Kruger’s report, whether the one-fluoroscopic (0.27 mSv) or the two-fluoroscopic technique (0.25 mSv) was used in the current study [6]. They assessed 36 PV procedures using the two-fluoroscopic technique. The average whole body dose (at the level of the operator’s chest) per vertebra was 1.44 mSv. Several different fluoroscopic modes were evaluated in their study: continuous fluoroscopy, high-level fluoroscopy and pulsed fluoroscopy. We agreed with their conclusions regarding the pulsed mode and we used the pulsed fluoroscopic mode. We found that it provided adequate cement image quality while limiting radiation exposure for the majority of patients undergoing PV.

**I**nstead of continuous imaging, we used intermittent imaging at every 2–3 turns of syringe compressor (0.2 ml/turn) during PV, not only to localize the entry point, but also during cement delivery [19]. In the current study, the use of intermittent imaging may decrease radiation dose, as compared with the use of continuous imaging. However, we consider blind deposition of cement to be potentially dangerous between images, therefore, we took an image at only one turn of the syringe compressor whenever cement was close to the posterior wall of the vertebral body. We stopped any procedure involving cement delivery if cement reached the posterior vertebral wall or entered the paravertebral veins.

**O**ur study had several limitations. Our sample size was small although there was a significant difference in radiation dose to the patients between the two groups. Second, theoretically, the radiation dose positively correlates with radiation exposure time. We did not measure the radiation exposure time and were not able to conclude whether or not the decreased radiation dose, using the two-fluoroscopic technique, resulted from the shorter radiation exposure time.

## Conclusions

In conclusion, the two-fluoroscopic technique during PV provides not only a shorter operative time but also is performed with less radiation dose to the patient compared to the one-fluoroscopic technique. There was no significant difference between the one- and two- fluoroscopic technique with regards to radiation exposure to the operator.

## Competing interests

The authors declare that they have no competing interests.

## Authors' contributions

Y–YL conceived of the design of the study, analyzed the radiographic measurements, drafted the manuscript, and performed all surgery. T–JH participated in the design of the study and coordinated the research groups. C–CC participated in the study. M–HW participated in the study. C-YL participated in the study. All authors read and approved the final manuscript.

## Pre-publication history

The pre-publication history for this paper can be accessed here:

http://www.biomedcentral.com/1471-2474/14/38/prepub
